# Editorial: Exploration of dietary correlates of conspiratorial thinking

**DOI:** 10.3389/fpsyg.2023.1205350

**Published:** 2023-06-22

**Authors:** Reza Rastmanesh, Neil Dagnall, Guoyan Wang

**Affiliations:** ^1^American Physical Society, College Park, MD, United States; ^2^Department of Psychology, Manchester Metropolitan University, Manchester, United Kingdom; ^3^School of Communication, Soochow University, Suzhou, China

**Keywords:** conspiratorial thinking, paranoia, dietary patterns, nutrient deficiencies, conspiracy theories

While scholarly interest in the origins, purpose, and consequences of conspiratorial thinking and endorsement of concomitant theories has an established tradition in psychological, work assessing the effects of conspiratorial thinking on general and individual diet-related perceptions, attitudes, and behaviors remains limited. Acknowledging this gap in the literature, the editors encouraged authors to highlight relevant developments and contemporary contributions within and around the topic domain. This was necessary since eating healthy foods and consuming drinks in the right proportions and amounts is vital to physical and psychological wellbeing. Accordingly, information that undermines health dietary patterns is potentially harmful to both individuals and society. In this context, conspiracy theories are an important source of inaccurate information that can inappropriately influence diet and detrimentally affect health.

Although there is no single, consensually agreed definition of conspiratorial thinking, commonly used delineations embody canonical themes. These include, but are not restricted to, exploitation of power, collusion, intention, clandestineness, deception, control, manipulation, and premeditation. These themes combine so that conspiratorial thinking reflects the belief that powerful individuals/groups, through exploitation, secretly enact actions to achieve, predetermined nefarious goals. In extreme cases, conspiratorial thinking predominates as a prevailing worldview, where high-order beliefs (e.g., mistrust of authority), askew perception of the world. Although not all conspiracies are false (e.g., Watergate, MKUltra, and Operation Northwoods), theories by definition typically convey false information as truth, or possibility.

In this regard, validation of mis (inaccurate) and/or dis(deliberate incorrect) information can adversely affect healthy life choices and habits. Examples include, theories related to genetically modified foods, sugar consumption, light/low fat products, and relationships between calorie burning and exercise. Despite evidence of a link between conspiratorial thinking and resistance to engage with public health communications, as evidenced during the COVID-19 pandemic, few studies to date have considered the influence of conspiratorial thinking on diet. Noting this, the present Research Topic asked for contributions addressing this issue and related areas. The outcome was a research collection that comprised research on conspiratorial thinking/theory endorsement, individual differences, and eating/diet/food choices.

With reference to conspiratorial thinking, submitted articles examined the theoretical nature of ideation and its application to diet-related issues. Considering contributions in turn, Franks et al. examined the monological nature of conspiracy acceptance. This is the view that belief in one theory correlates positively with endorsement of others. Franks et al. did this by reconstructing a conspiracy worldview. This approach identified novel features of conspiracy endorsement (e.g., community and personal journey of conversion). Orosz et al. found that rational and ridiculing arguments reduced CT endorsement, and the perceived intelligence and competence of the source of belief-reduction information contributed to the success of the reduction strategy. Prichard and Christman investigated factors associated with a lack of concern about COVID-19 and belief that China was responsible for the virus. Authoritarianism was related with less concern about the virus and Authoritarianism and Conspiracy Beliefs accounted for unique variance in blame on China for the virus. Applied to diet, these article signify that conspiratorial thinking is elaborate and that strategies to increase accurate awareness of health-related matters need to acknowledge this. Particularly, rational arguments that target the connection between the object of belief and its characteristics in a subtle non-confrontational manner, which are presented by credible sources are most likely to reduce conspiratorial thinking.

Articles examining conspiratorial thinking in specific diet-related contexts also produced valuable outcomes. Du et al. found that information exposure was directly connected to attitudes about genetically modified organisms (GMO). Beliefs in conspiracy theories also played a mediating role. Specifically, unofficial information reinforced beliefs in conspiracy theories and stronger beliefs reduced willingness to consume GMO. In contrast, exposure to official information weakened people's beliefs in conspiracy theories and increased their willingness to consume GMO foods. Additionally, knowledge had a moderating role. Objective knowledge reduced conspiracy beliefs, whereas self-assessment enhanced them. Also on GMO, Yang reported that citizen science communicators and scientist science communicators employ different discourse strategies to convey oppositional attitudes to GMOs conspiracy theories. Jedinger found that conspiracy mentality correlated with the perceived threat posed by foreign trade and opposition to international trade. Collectively, these articles illustrate that exposure to conspiracies and contextualization of allied thinking has an important influence on perception of food types and the sourcing of provisions.

In terms of individual differences this Research Topic produced several articles focusing diet and eating. Sariyska et al. investigated how the consumption of animal products was related to dietary habits, primary emotional systems, and dark triad personality traits. Uccula et al. reported that in a potentially threatening situation, there was an association between attachment orientation and preference to use care or food to regulate their negative emotions. Zhang et al. outlines how general and food-specific inhibitory control mechanisms moderate the predictive relationship between automatic attention and food choices. Nettle discusses the notion that individuals of lower socioeconomic position behave and feel as they do because of relative hunger and concludes that hunger is an important mediator between socioeconomic variables and behavioral/psychological outcomes. Cantarero et al. investigated feedback to a dish poorly prepared by a stranger. Outcomes designated that participants were most likely to opt for prosocial lies (i.e., overly positive feedback) when the stranger cared about cooking and was very sensitive to negative feedback.

These papers investigators with a better understanding of how individual differences influence food and nutritional choices. This information is useful to the topic of conspiratorial thinking and diet since it informs possibilities for subsequent research. For instance, future work could assess whether vegans/vegetarians (vs. omnivores) are more susceptible to eating choice based conspiracies and determine whether the observed relationship is influenced by dark triad personality traits. Additionally, studies could examine whether attachment orientation and negative emotions increase the tendency to endorse food, diet, and nutrition based conspiracy theories. There is certainly pertinent extant literature to suggest that areas such as these would produce finding that extend understanding of dietary correlates of conspiratorial thinking.

Within the Research Topic, three articles focused on eating choices and habits. These overlapped with the submissions on individual differences, and similarly suggested useful investigative avenues for ensuing scholarly. Huang et al. found that consumers were susceptible to the influence of targeted marketing strategies for foods with a low-calorie claim. Vestergren and Uysal, following a systematic review of veganism and sustainable diet/lifestyle between 2010 and 2021, identified important themes such as treating all non-meat eaters as a homogeneous group and lack of processes underlying emergence and endurance of veganism, which have limited understanding of veganism and vegan identity. Finally, Moynihan et al. observed that boredom predicted maladaptive and adaptive eating behaviors as a function of the need to distance from the experience of boredom.

The papers in this Research Topic reinforce the need for concerted work in the area of dietary correlates of conspiratorial thinking. Whilst these contributions provide novel conceptual insights and recommend ways to progress the Research Topic, the area despite its social importance, remains relatively under researched. Moreover, within the domain there is a need for greater focus and coherence. Presently, due to breadth, investigation is diffuse and only peripherally connected. Nonetheless, the work in this Research Topic is valuable since in order to develop strategies to counteract the negative effects of conspiratorial thinking on diet, researchers first need to understand allied psychological processes and contextual constraints.

## Author contributions

RR wrote the Research Topic and contributed to the draft of editorial. ND wrote the editorial and contributed to the editorship. GW contributed to editorship. All authors contributed to the article and approved the submitted version.

